# Prognostic impact of the level of neck metastasis in oral cancer patients

**DOI:** 10.5935/1808-8694.20120027

**Published:** 2015-10-20

**Authors:** Hugo Fontan Köhler, Luiz Paulo Kowalski

**Affiliations:** a MD (Former resident MD in the Department of Head and Neck Surgery at A. C. Camargo Hospital); b Assistant Professor (Director of the Department of Head and Neck Surgery at A. C. Camargo Hospital)

**Keywords:** head and neck neoplasms, mouth neoplasms, neck dissection, prognosis, survival rate

## Abstract

Neck staging in oral cancer depends on the number of compromised nodes, their size and side of occurrence.

**Objective:**

This paper aims to evaluate risk factors for metastatic nodes in levels IV/V and their prognostic impact on patients with oral carcinoma.

**Method:**

Retrospective study. Inclusion criteria: pathologist's diagnosis of squamous cell carcinoma, primary tumor in the lower oral cavity, no extension into extraoral sites, no previous treatment, synchronous neck dissection and presence of metastatic nodes. Risk factors for metastasis were evaluated through logistic regression and disease-specific survival and recurrence by survival analysis. Classificatory analysis was performed through recursive partitioning.

**Results:**

307 patients met the inclusion criteria. Univariate logistic regression identified pN stage, vascular invasion, and multiple metastatic nodes as risk factors for metastases in levels IV/V. Multivariate analysis found vascular invasion and multiple metastatic nodes were significant. Survival analysis revealed pT, pN, neural infiltration, vascular invasion, number of metastatic nodes, metastases in levels IV/V, and node ratio were significant factors. In multivariate survival analysis, pT, pN, vascular invasion and metastases in levels IV/V were significant. Classificatory analysis showed that pN is non-significant in patients with level IV/V metastases.

**Conclusion:**

The occurrence of metastases in levels IV/V was significant for disease-specific survival.

## INTRODUCTION

Squamous cell carcinomas (SCC) of the oral cavity are categorized in different stages to reflect prognostic factors and determine the most adequate standard course of therapy. Tumor staging looks into the characteristics of the primary tumor along with node and systemic metastases^1^. Neck lymph nodes are usually the first site affected by metastases in patients with high digestive tract SCC. Presence of metastasis is one of the most relevant prognostic factors for patients with oral SCC^2^.

Neck staging in patients with high digestive tract tumors is standard for most primary tumor sites and is based on the number of metastatic nodes, the size of the largest node, and the side of the involved node in relation to the primary tumor. Depending on the combination of these factors, patients are assigned to one of six stages^3^. However, this system does not cover all prognostic factors known to be significant, such as the presence of extracapsular tumor extension or lymph node density^4^, ^5^. The staging system's prognostic role has also been criticized due to the limitations it poses mainly in patients submitted to adjuvant therapy, for whom its discriminatory power is significantly diminished^6^.

Neck lymph nodes are grouped in levels related to well defined anatomic regions of the neck. Tumor involvement of neck nodes does not occur randomly. Patterns of involvement can be predicted as a function of the primary tumor site, as described in the literature, and used to aid in selective neck dissection (ND) procedures^7^. Node metastases in oral SCC occur initially on levels I to III, and that is the rationale for selective ND involving these levels and, further on, they reach levels IV and V^8^.

This paper aimed to analyze the risk factors for the presence of node metastasis on levels IV/V and their impact on the disease-specific survival of patients with oral carcinoma.

## METHOD

This study enrolled patients treated consecutively at one institution from January 1985 to December 2005. Inclusion criteria: confirmed diagnosis of SCC by histopathology testing, primary tumor located in the lower oral cavity, no tumor extension onto extraoral sites or onto the oropharynx, no previous treatment, synchronous neck dissection, and presence of metastatic nodes on surgical specimen. Exclusion criteria: patients with tumors other than SCC as confirmed by histopathology testing, tumors extending to the skin, oropharynx or other sites, patients submitted to previous treatment, primary tumor surgery without neck dissection, patients without metastatic nodes on pathology testing (pN0), patients receiving radiotherapy/chemoradiotherapy, and patients with diagnosed systemic metastasis. The staging of all patients was updated as per the standard procedure described in 2009^1^. This study was approved by the Institutional Research Ethics Committee (permit n^o^ 571/2007).

Statistical analysis was performed on software program Stata 12.1 for MacOS. Categorical variables were described by their frequencies of occurrence, whereas continuous variables were described in terms of their mean values and standard deviations (SD). Logistic regression was used to correlate risk factors to the presence of level IV/V metastases. Survival analysis was done based on the Kaplan-Meier and Nelson-Aalen curves, combined to the Cox model. Classificatory analysis was performed by recursive partitioning, with divisions with a minimum of five cases and a cutoff value of 0.05. Percentages were rounded up to two decimal fractions and continuous variables to three decimal fractions. *P*-values equal to or smaller than 0.05 were deemed statistically significant.

## RESULTS

Three-hundred and seven consecutive patients were included in this study. The sample had 266 males (86.64%) and 41 females (13.36%). Age at diagnosis ranged between 22 and 85 years (mean: 56.13 years; SD: 10.50 years). Primary tumor sites can be seen on Table 1. Primary tumors were staged as pT1 in nine patients (2.93%), pT2 in 112 patients (34.68%), pT3 in 99 patients (32.25%) and pT4a in 87 patients (28.34%). Neck dissection was performed in all patients simultaneously to primary tumor ablation. ND on levels I-IV (supraomohyoid extended to level IV) was performed on 177 patients (57.65%), modified radical ND in 85 patients (27.69%), and classic radical ND in 45 patients (14.66%). Ninety-three patients (30.29%) underwent bilateral ND. The number of retrieved nodes on ipsilateral dissections ranged from four to 116 (mean: 45.7 nodes; SD: 16.42 nodes), while in the contralateral neck this number ranged from six to 73 nodes (mean: 29.28; SD:16.99). The number of metastatic nodes ranged from one to 47 in the ipsilateral neck and from none to nine on the contralateral neck. Node density ranged from 0.011 to 0.979 (mean: 0.082; SD: 0.011). Clinical and pathological neck staging and the distribution of stages can be seen on Table 2. Table 3 shows the number and percentage of patients with level IV/V metastases for each cN and pN stage. In our series, in the group of patients with one single metastatic node (pN1/pN2a), three (2.91%) had level IV/V involvement. Primary tumor vascular invasion was found in 214 patients (72.79%) and neural infiltration in 141 subjects (49.13%). Patients were followed for 6.3 to 298.2 months (mean: 44.9 months; SD: 22.4 months). At the end of the follow-up period, 164 patients (53.42%) had died of neoplastic disease.

**Table 1:** Primary tumor sites of the patients included in this series.

Primary site	Number of patients	Percent
Oral tongue	132	43.00%
Mouth floor	63	20.52%
Retromolar trigone	85	27.69%
Lower gum ridge	23	7.49%
Cheek mucosa	4	1.30%
Total	307	100%

**Table 2:** Distribution of patients as a function of neck clinical and pathology staging.

	pN1	pN2a	pN2b	pN2c	pN3	Total
cN0	42	3	44	7	0	96
cN1	39	2	53	14	0	108
cN2a	4	1	17	1	2	25
cN2b	8	0	31	8	1	48
cN2c	2	0	1	12	0	15
cN3	1	1	8	4	1	15
Total	96	7	154	46	4	307

**Table 3:** Distribution of patients with level IV/V metastasis s a function of neck pathology staging.

cN	Number of patients	Level IV/V metastasis(%)	pN	Number of patients	Level IV/V metastasis(%)
cN0	96	7 (7.29)	-	-	-
cN1	108	10 (9.25)	pN1	96	2 (2.08)
cN2a	25	3 (12.00)	pN2a	6	1 (16.67)
cN2b	48	9 (18.75)	pN2b	132	22 (16.67)
cN2c	15	1 (6.67)	pN2c	37	9 (24.32)
cN3	15	4 (26.67)	pN3	4	0 (0)

Univariate analysis elicited the following as risk factors connected to the presence of level IV/V metastasis: pN stage (*p* = 0.002), vascular invasion *(p* = 0.021), and multiple involved nodes (*p* < 0.001). In multivariate analysis, vascular invasion *(p* = 0.045) and multiple involved nodes (*p* < 0.001) had statistical significance (Table 4).

**Table 4:** Multivariate analysis by logistic regression of the risk factor for level IV/V metastasis.

Variable	Odds ratio	95% CI	*p*-value
Vascular invasion	3.877	1.032-14.574	0.045
Involved nodes	1.484	1.287-1.710	< 0.001

CI: Confidence Interval.

**Figure 1:**
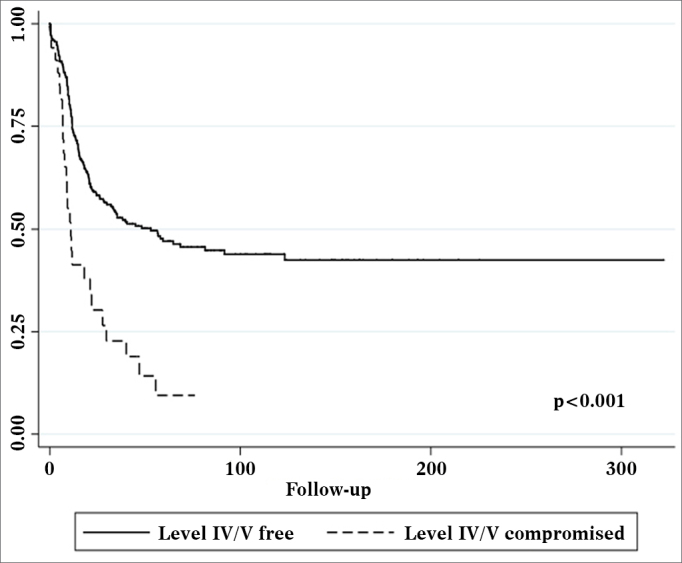
Kaplan-Meier survival curve for patients with and without level IV/V metastatic nodes.

Univariate survival analysis revealed that pT and pN stages, vascular invasion, neural infiltration, number of metastatic nodes, node ratio, and presence of metastatic nodes on levels IV and V were statistically significant (Table 5). These variables were included in a stepwise approach to multivariate analysis, which revealed that pT stage, pN stage, vascular invasion, and node metastases on levels IV and V were statistically significant (Table 6). Patients with metastasis on levels IV and V had worse disease-specific survival than those with metastatic disease limited to levels I to III (Figure 1).

**Table 5:** Significant variables identified in disease-specific survival univariate analysis.

Variable	Relative risk	95% CI	*p*-value
pT stage	1.618	1.342-1.950	< 0.001
pN stage	1.228	1.066-1.414	0.004
Level IV/V metastasis	2.624	1.717-4.008	< 0.001
Vascular invasion	1.695	1.142-2.518	0.009
Neural infiltration	1.613	1.165-2.232	0.004
Number of metastatic nodes	1.037	1.013-1.061	0.002
Node ratio	5.278	1.644-16.949	0.005

CI: Confidence Interval.

**Table 6:** Disease-specifc survival multivariate analysis.

Variable	Relative risk	95% CI	*p*-value
pT stage	1.618	1.326-1.975	< 0.001
Level 4/5 metastasis	1.962	1.249-3.080	< 0.001
Vascular invasion	1.458	1.087-2.191	0.037
pN stage	1.447	1.041-2.011	0.028

CI: Confdence Interval.

**Figure 2:**
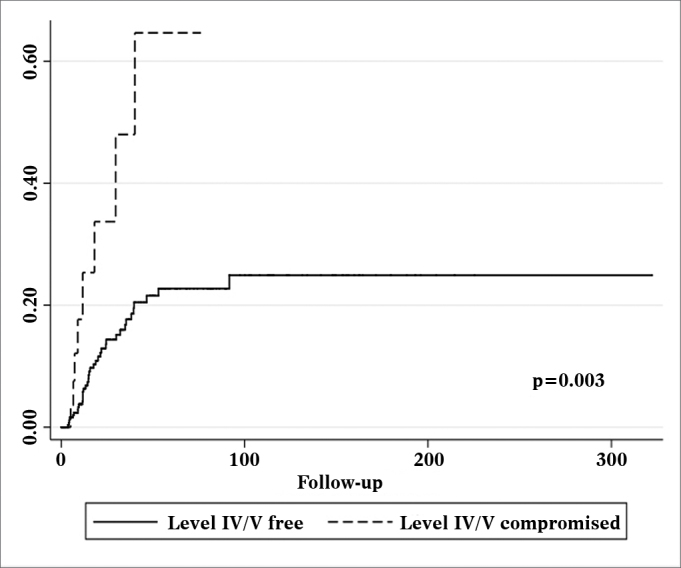
Nelson-Aalen cumulative risk curve for occurrence of systemic metastasis correlated with the presence of level IV/V metastatic nodes.

**Figure 3:**
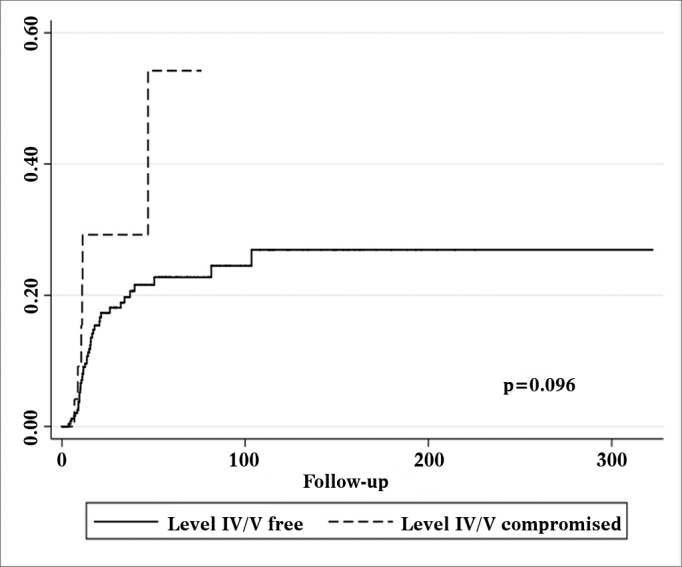
Nelson-Aalen cumulative risk curve for occurrence of relapsing neck disease correlated with the presence of level IV/V metastatic nodes.

Patients with metastatic nodes on levels IV/V were at significantly higher risk for systemic metastasis than those without neoplastic involvement in these levels (relative risk (RR): 3.182; 95% confidence interval (CI): 1.472-6.877; *p* = 0.003, Figure 2). Nonetheless, neck recurrence was not affected by metastases on levels IV/V (RR: 2.079; 95% CI: 0.879-4.915; *p* = 0.096, Figure 3).

**Figure 4:**
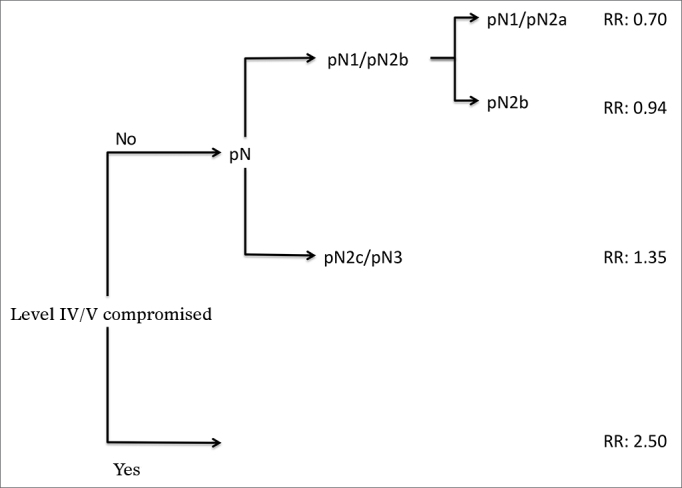
Classifcatory analysis by patient recursive partitioning (RR: Relative Risk). All partitions with *p* < 0.05.

Analysis by recursive partitioning including the presence of neck metastasis on levels IV/V and pN stage revealed that the latter was not significant for patients with metastasis on levels IV/V, although it was significant for patients with neoplastic involvement on levels I/III (Figure 4). When patients were stratified as a function of the presence of metastasis on levels IV/V, prognostic factors varied between groups and it was not possible to determine which related to the group with neoplastic involvement on levels IV/V (Table 7).

**Table 7:** Survival multivariate analysis after patients were stratifed according to the presence r absence of level IV/V metastatic nodes.

	No level IV/V metastasis	Present level IV/V metastasis
Variable	RR (95% CI), *p*-value	RR (95% CI), *p*-value
pT stage	1.654 (1.332-2.053), < 0.001	1.200 (0.555-2.591), 0.642
pN stage	1.325 (1.089-1.821), 0.015	0.870 (0.402-1.887), 0.725
Vascular invasion	1.583 (1.036-2.418), 0.034	0.872 (0.168-4.532), 0.870

RR: Relative Risk; CI: Confdence Interval.

## DISCUSSION

Cancer patient staging is done to estimate prognosis and help define the need for adjuvant therapy in surgery patients. For patients with oral SCC, the pN stage is highly significant for disease-specific survival and should be used to stratify these patients in trials due to its relevant prognostic impact^9^.

The extension of selective ND in oral SCC patients to include levels IV and V has been discussed in the literature. A paper comparing neck relapse rates in patients submitted to selective and radical ND revealed similar results for both approaches, and the authors concluded that the removal of nodes on levels I to III was sufficient^10^. No cases of isolated level IV involvement were seen in 81 oral SCC patients submitted to selective ND, thus indicating to the authors that node removal was not necessary in cN0 patients. However, the authors stressed that the metastasis distribution pattern is less predictable in cN+ patients, although no isolated level IV and V metastases were found. When cN0 and cN+ patients were compared, the rates of metastasis on level IV were found to be 0% and 9% respectively^11^. Such finding was not confirmed in our series, as even cN0 patients had level IV/V involvement in 7.29% of the cases. Likewise, another retrospective series failed to find isolated level V node metastasis in patients with high digestive tract SCC and reported level V involvement in 15.1% of pN+ patients. According to the authors, such finding would not justify patient elective management^12^. However, in an analysis done on 119 elective ND procedures, the authors found approximately 5% of isolated level IV metastasis with no involvement in other levels. The authors suggested that level IV be routinely included in the neck dissections of cN0 and cN1 patients and that level V be approached for all other patients^13^.

This data is supported by another series, in which an incidence rate of 4.6% of level IV metastasis was reported after elective ND in oral SCC patients, with the exception of the oral tongue, in which case the incidence rate was 5.6%. There was no statistically significant difference on the neck relapse rates of patients submitted to ND for levels I-III and I-IV when postoperative radiotherapy was offered. Thus, the authors suggested that the removal of level IV nodes would not benefit patients^14^. Another series including oral and oropharyngeal primary tumors considered that managing level V was unnecessary in pN1 and pN2 patients due to the low incidence rates of metastasis observed in this area. The occurrence of level V metastasis in this series was significantly correlated to N stages above pN2b and to the presence of metastasis in multiple levels^15^. In our series, the incidence rate of metastasis on levels IV/V was 2.91%, considering patients with one involved node. This is a small number when compared to the threshold of 20% of risk of metastasis traditionally accepted in the indication for elective ND^16^. Additionally, the risk factors for the presence of level V metastasis were related to pathological characteristics available only on the final pathology tests.

The impact of the presence of metastasis on the survival of these patients is a topic that requires more discussion in the literature. An analysis of patients submitted to selective ND revealed low relapse rates on level V nodes. In such series it was evident that the presence of extracapsular extension and multiple metastatic nodes were the most relevant prognostic factors in the neck^17^. A retrospective series with pN2 patients showed that the presence of node metastasis on levels IV/V was a significant prognostic factor for patient survival^18^. In our series this prognostic factor was also assessed for pN1 patients and proved to be statistically significant for them as well. Additionally, the presence of level IV/V metastasis was found to overwhelm all other parameters related to the N stage in terms of prognostic status in a classificatory analysis. Therefore, this seems to be the main prognostic trait for patients with node involvement.

## CONCLUSION

The occurrence of metastasis on levels IV/V correlates to the pathological characteristics of the primary tumor. Isolated occurrence of level IV/V metastasis is rare. Neoplastic involvement in levels IV and V is in itself an important prognostic factor for patients with oral squamous cell carcinoma and impacts disease-specific survival, but does not affect neck relapse rates. This fact poses relevant impact upon the occurrence of systemic metastasis and must be taken into account in the indication of adjuvant therapy and, possibly, in the staging of these tumors.
